# Evaluating Convolutional Neural Networks as a Method of EEG–EMG Fusion

**DOI:** 10.3389/fnbot.2021.692183

**Published:** 2021-11-23

**Authors:** Jacob Tryon, Ana Luisa Trejos

**Affiliations:** ^1^School of Biomedical Engineering, Western University, London, ON, Canada; ^2^Department of Electrical and Computer Engineering, Western University, London, ON, Canada

**Keywords:** convolutional neural networks, EEG signals, EMG signals, human-machine interfaces, sensor fusion

## Abstract

Wearable robotic exoskeletons have emerged as an exciting new treatment tool for disorders affecting mobility; however, the human–machine interface, used by the patient for device control, requires further improvement before robotic assistance and rehabilitation can be widely adopted. One method, made possible through advancements in machine learning technology, is the use of bioelectrical signals, such as electroencephalography (EEG) and electromyography (EMG), to classify the user's actions and intentions. While classification using these signals has been demonstrated for many relevant control tasks, such as motion intention detection and gesture recognition, challenges in decoding the bioelectrical signals have caused researchers to seek methods for improving the accuracy of these models. One such method is the use of EEG–EMG fusion, creating a classification model that decodes information from both EEG and EMG signals simultaneously to increase the amount of available information. So far, EEG–EMG fusion has been implemented using traditional machine learning methods that rely on manual feature extraction; however, new machine learning methods have emerged that can automatically extract relevant information from a dataset, which may prove beneficial during EEG–EMG fusion. In this study, Convolutional Neural Network (CNN) models were developed using combined EEG–EMG inputs to determine if they have potential as a method of EEG–EMG fusion that automatically extracts relevant information from both signals simultaneously. EEG and EMG signals were recorded during elbow flexion–extension and used to develop CNN models based on time–frequency (spectrogram) and time (filtered signal) domain image inputs. The results show a mean accuracy of 80.51 ± 8.07% for a three-class output (33.33% chance level), with an F-score of 80.74%, using time–frequency domain-based models. This work demonstrates the viability of CNNs as a new method of EEG–EMG fusion and evaluates different signal representations to determine the best implementation of a combined EEG–EMG CNN. It leverages modern machine learning methods to advance EEG–EMG fusion, which will ultimately lead to improvements in the usability of wearable robotic exoskeletons.

## 1. Introduction

The field of assistive and rehabilitation robotics is rapidly growing, seeking to leverage modern technological advancements to help patients suffering from mobility issues to restore their quality of life. With musculoskeletal disorders being the largest contributor to worldwide disability (World Health Organization, 2019), there is a large market for such devices to help supplement the treatment provided by traditional therapy. Wearable upper-limb robotic exoskeletons, in particular, present a promising option for rehabilitation and assistance, since the patient can use the device during daily life to help assist with tasks, and they are not constrained to a single location during rehabilitation therapy. These devices, however, are still limited in their use, and one reason for this is that further development is required to advance the intelligence of the control methods used in these systems (Desplenter et al., 2020). The devices should be controlled in such a way that their use feels natural and comfortable for the user, regardless of the task being performed.

One popularly explored method to achieve this is the use of bioelectrical signals, produced by the body during motion, to directly control the wearable robotic exoskeletons by detecting the user's motion intention and movement activity based on the information encoded in these signals. Two popularly used bioelectrical signals are electroencephalography (EEG), recorded from brain activity, and electromyography (EMG), recorded from muscle activity (Sawangjai et al., 2020; Leelaarporn et al., 2021). These signals are measured using electrodes on the skin and can be decoded (often through the use of machine learning techniques) to facilitate the control of wearable robotic systems. Typically, devices will only make use of one bioelectrical signal type at a time (Desplenter et al., 2020); however, studies have emerged that have shown that the simultaneous use of EEG and EMG together can improve system performance (Leeb et al., 2011; Dulantha Lalitharatne et al., 2013; Xie et al., 2013; Novak and Riener, 2015; Li et al., 2017; Wöhrle et al., 2017; Loopez-Larraz et al., 2018; Sbargoud et al., 2019; Tryon et al., 2019; Gordleeva et al., 2020; Tortora et al., 2020; Tryon and Trejos, 2021). It has been shown that EEG–EMG fusion can improve classification accuracy as well as reliability, by leveraging the benefits of both signal types simultaneously. An example of this is the use of EEG–EMG fusion as a method to combat the effect of muscle fatigue on system performance. Studies have shown that EEG–EMG fusion models can maintain sufficient accuracy even during EMG signal attenuation brought on by muscle fatigue (Leeb et al., 2011; Tortora et al., 2020), demonstrating the increased reliability that can be obtained through the use of multiple signal types simultaneously. Typically, EEG–EMG fusion is used with machine learning to perform a classification task relevant to the control of a robotic exoskeleton device (for example, motion intention detection). A commonly used method to incorporate EEG–EMG fusion into machine-learning-based classification is to perform EEG–EMG fusion at the decision level, meaning that two classifiers are trained (one for EEG, one for EMG) and their outputs are combined using various techniques (Leeb et al., 2011; Wöhrle et al., 2017; Sbargoud et al., 2019; Tryon et al., 2019; Gordleeva et al., 2020; Tortora et al., 2020; Tryon and Trejos, 2021). Use of this method has been successfully demonstrated for tasks such as motion classification, for example, obtaining an accuracy of 92.0% while outperforming EEG and EMG only models (Leeb et al., 2011). Some examples exist of EEG–EMG fusion happening at the input level, meaning that EEG and EMG features are used simultaneously to train one classifier (Xie et al., 2013; Li et al., 2017; Loopez-Larraz et al., 2018; Tryon et al., 2019; Gordleeva et al., 2020; Tryon and Trejos, 2021). Studies that focus on this technique have been able to show accuracies similar to decision-level fusion studies, in one example obtaining an accuracy of 91.7% using a single classifier for gesture recognition (Li et al., 2017); however, when compared with decision-level fusion in the same study, input-level fusion is often found to yield poorer results (Gordleeva et al., 2020; Tryon and Trejos, 2021).

Despite promising results, further development is needed for EEG–EMG fusion techniques to improve their viability for use in wearable robotic systems. The vast majority of EEG–EMG fusion has been done using traditional machine learning methods that rely on manual feature extraction before training the classifier. Recently, new machine learning methods (often referred to as deep learning) have emerged that are capable of automatically extracting feature information from inputs. One of the most notable implementations of deep learning is the Convolutional Neural Network (CNN). These CNN models, originally developed for the image processing domain, work by using convolution layers that extract information from around an image before feeding it into traditional neural network layers (called Fully Connected layers). The model is not only able to learn patterns from within the data, like traditional machine learning, but also automatically learn what relevant information to extract from the input (instead of relying on the user to specify this manually through selection of appropriate features). The success of CNN classifiers have caused them to move beyond the image processing domain into other areas, with bioelectical signal classification being one of them. For both EEG (Roy et al., 2019) and EMG (Phinyomark and Scheme, 2018), CNNs are the most popularly used deep learning technique. Many studies have shown great results when using CNNs with EEG (Schirrmeister et al., 2017; Wang et al., 2018; Amin et al., 2019; Chaudhary et al., 2019; Dai et al., 2019; Ditthapron et al., 2019; Li et al., 2019; Tayeb et al., 2019; Zhang et al., 2019; Zhao et al., 2019; Tang et al., 2020; Wilaiprasitporn et al., 2020) and EMG (Atzori et al., 2016; Zhai et al., 2017; Ameri et al., 2018; Ding et al., 2018; Xia et al., 2018; Zia ur Rehman et al., 2018; Côté-Allard et al., 2019; Duan et al., 2019; Chen et al., 2020; Fang et al., 2021) signals and have been able to perform many tasks relevant to control, such as hand gesture recognition or the implementation of a Brain Computer Interface, based on Motor Imagery, to send device commands. A brief selection of CNN-based EEG/EMG literature with control-relevant tasks can be seen in Table 1. Despite the popularity of CNN models in EEG and EMG literature, the area of EEG–EMG fusion has yet to widely adopt the use of this technique. One study showed that CNNs can be used to fuse EEG and EMG (along with Electrooculography, known as EOG) for sleep stage classification (Banluesombatkul et al., 2021); however, it remains to be seen how an EEG–EMG CNN classifier would perform if used during motion tasks that are relevant for control of assistive and rehabilitation robots. It is possible that the CNN model may extract information about the relationship between the two signals, recorded while the user is moving, that is not currently captured using manually selected features that have been combined for input-level fusion. There is further evidence of CNNs being able to extract information from both EEG and EMG, since a study was done where transfer learning (initially training a classifier for one type of data, then using that classifier with a different set of data) was performed between EEG and EMG datasets with CNNs. The study found that transfer learning was possible between the two signal types to classify concentration levels (EEG) and hand gestures (EMG) (Bird et al., 2020). This may indicate that there is a relationship between the bioelectrical signals that a CNN can detect; therefore, more experimentation is needed to further evaluate CNNs as a method of input level EEG–EMG fusion.

**Table 1:** A summary of select literature examples using CNN models with EEG or EMG signals.

**Signal type**	**Application**	**Reference**
EEG	2 Class motor imagery (e.g., left hand, right hand)	Wang et al., 2018; Chaudhary et al., 2019; Dai et al., 2019; Tayeb et al., 2019; Tang et al., 2020
	4 Class motor imagery (e.g., left hand, right hand, feet, tongue)	Schirrmeister et al., 2017; Amin et al., 2019; Li et al., 2019; Xu et al., 2019; Zhang et al., 2019
	6 Class motor imagery (i.e., elbow flexion/extension, forearm supination/pronation, hand open/close)	Zhao et al., 2019
	Person identification	Wilaiprasitporn et al., 2020
	P300 Classification	Ditthapron et al., 2019
EMG	Hand gesture classification	Zhai et al., 2017; Ding et al., 2018; Zia ur Rehman et al., 2018; Côté-Allard et al., 2019; Duan et al., 2019; Chen et al., 2020; Fang et al., 2021
	Wrist movement classification	Ameri et al., 2018
	Hand movement/Gesture classification	Atzori et al., 2016; Zhai et al., 2017
	Hand position estimation	Xia et al., 2018

*The references are grouped by signal type and application*.

The objective of this work was to evaluate CNNs as a method of EEG–EMG fusion, and to perform an analysis of the feasibility of this technique when used for a classification task relevant to the control of assistive and rehabilitation robots. Multiple methods of representing and combing the EEG/EMG signals at the input level were investigated to see which method of EEG–EMG fusion would provide the best performance within the CNN classifier. This work provides an example of EEG–EMG fusion happening within the CNN classifier, and highlights the most promising methods to use for further development. To facilitate this evaluation, it was decided to train models to classify task weight during dynamic elbow flexion–extension motion. Task weight is the weight a user is holding during movement. This is relevant to the control of wearable robotic exoskeletons during assistance and rehabilitation because the presence of an external weight can affect the stability of a bioelectrical-signal-based control system (Desplenter and Trejos, 2018; Desplenter et al., 2020), as well as the accuracy of control-relevant classification tasks, such as hand gesture recognition (Teh and Hargrove, 2020). These control systems are often tuned for specific movement conditions; hence, being able to detect what the user is holding, will allow the control system to dynamically adapt to the new disturbance and provide more robust performance as the user changes tasks during their daily life. Measuring task weight during dynamic elbow flexion–extension motion provides a more realistic evaluation of the models (as opposed to isometric muscle contraction), since the end goal of EEG–EMG fusion is to use it within a wearable robotic exoskeleton during different motions. Dynamic movement, as well as the more indirect force measurement of task weight, can greatly increase the challenge of performing classification tasks with EEG and EMG signals; hence, EEG–EMG fusion provides a good opportunity to investigate potential improvements to address these limitations. The authors' previous work evaluated EEG–EMG fusion methods for task weight classification, and obtained accuracies of 83.01% using decision-level fusion and 80.75% using input-level fusion; however, this was done using fusion methods based on traditional machine learning classifiers with manual feature extraction (Tryon and Trejos, 2021). This paper focuses on evaluating CNN-based EEG–EMG fusion on the same classification task as a means of comparison.

## 2. Methods

### 2.1. Data Collection and Signal Processing

To develop EEG–EMG-fusion-based CNN models, a dataset of EEG and EMG signals were collected during elbow flexion–extension motion from 32 healthy subjects (mean age 24.9 ± 5.4 years) who were voluntarily recruited following approval from the Human Research Ethics Board at Western University (Project ID: 112023). The data obtained from these subjects were also used in previous studies by the authors (Tryon et al., 2019; Tryon and Trejos, 2021). The subjects were instructed to perform the motion at two speeds level (approximately 10°/s and 150°/s) and three weight levels (0 lbs, 3 lbs, 5 lbs), implementing a 2 × 3 full factorial repeated measures study design. This resulted in six combinations of weight and speed being recorded (each pairing referred to as a trial). The order in which the trials were performed was randomized for each subject to limit any potential biasing effects caused by the ordering of the speed/weight pairings. Within each trial, elbow flexion–extension motion was performed for three repetitions using the subject's dominant arm (30 right handed, 2 left handed), with a 3 s pause in-between repetitions. Between each trial, subjects were given a 1-min rest period. While performing the elbow flexion–extension motion, the subject would self-regulate their motion speed to achieve an approximation of the targeted speed. Subjects were instructed by the experimenter to count seconds while performing each elbow flexion–extension repetition such that a 30 s motion duration was obtained for the slow speed (10°/s) repetitions and a 2 s duration was obtained for the fast speed (150°/s) repetitions. Assuming a 150° range of motion, this resulted in approximately the desired speed for each targeted speed level, while still allowing the subject to move dynamically in an unrestricted manner.

During data collection, the EEG and EMG signals were recorded using an Intronix 2024F Physiological Amplifier System (Intronix Technologies, Bolton, Canada). Both EEG and EMG were sampled at 4,000 Hz and a ground electrode was placed over the elbow bone of the subject's non-dominant arm to act as the system ground for the differential amplifier. The sampling rate of the measurement system was fixed for all channels and could not be altered, which is why it was higher than necessary, particularly for the EEG signals. In an actual wearable robotic device, this sampling rate would be lower to reduce hardware demands.

To record EEG signals, wired gold-cup electrodes, filled with electrically conductive paste, were placed on the subject's scalp above the C3, C4, and Cz locations, as specified by the 10–20 International System. These locations were chosen for this study since they correspond with the motor cortex of the brain, and should provide relevant signal information during movement. Prior to placing the electrodes, the subject's scalp was cleaned at the location of electrode placement with an abrasive gel to ensure that a proper electrical connection was established. Signals were recorded using bipolar channels, configured for a reference montage, with the reference point being an ear-clip electrode attached to the subject's ear lobe. During recording, the EEG signals were filtered with a 0.5–100 Hz band pass filter built into the Intronix 2024F system. After recording, the EEG signals were filtered again in software using a 0.5–40 Hz band pass filter (3^*rd*^ order Butterworth) (Vaid et al., 2015).

To record EMG signals, bipolar electrodes were placed over the biceps and triceps of the subject's dominant arm, as specified by the SENIAM Project guidelines. These muscles were chosen for this study since they are two of the main muscles that contribute to elbow flexion–extension motion. Prior to electrode placement, the subject's skin at the location of electrode placement was cleaned using an alcohol swab. During recording, the EMG signals were filtered with the measurement system's built-in 20–500 Hz band pass filter. Following recording, the EMG signals had the DC offset removed and were filtered again with another 20–500 Hz band pass filter (4^*th*^ order Butterworth) (De Luca, 2002).

After filtering, the signals were segmented to remove the portions of the recording where the subject was not moving. This was done using markers that were placed at the beginning and end of the subject's movement. The markers were placed manually by the experimenter during data recording using an external trigger system. Synchronized video recordings of the subject moving were also recorded for verification.

**Figure 1:**
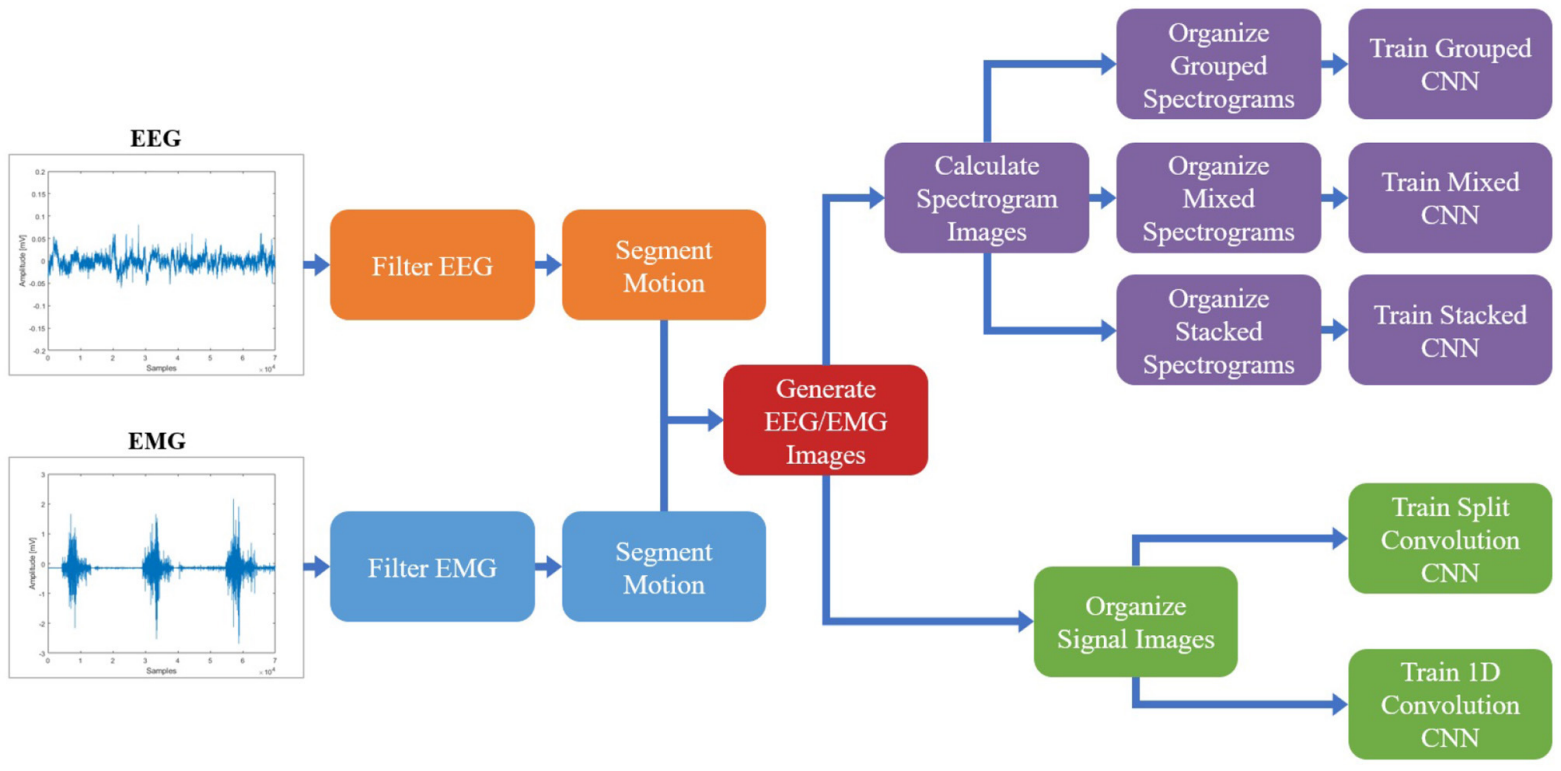
The protocol followed to process the EEG/EMG signals, generate the spectrogram and signal images, and train the CNN models using different EEG–EMG fusion methods. The top path (purple) shows the steps used to develop the CNN models based on spectrogram image inputs, while the bottom path (green) shows the steps used to develop the CNN models based on signal image inputs. For all EEG–EMG-fusion-based CNN model types (represented by the final step of all paths), an EEG and EMG only version was also trained, to provide a baseline comparison for evaluating EEG–EMG Fusion.

All signal processing and image generation was done offline using MATLAB 2019b with the Signal Processing Toolbox. An overview of the full data processing pipeline can be seen in Figure 1.

### 2.2. Image Generation

Once the EEG and EMG signals were processed and segmented, the next step was to convert the dataset into images that can act as suitable inputs to a CNN classifier. Since CNNs were developed initially as a tool for image recognition problems, their architecture relies on images as inputs; however, since an image is simply an array with a numerical value at each pixel location, it is possible to represent bioelectrical signals in such a way. In previous works that have used EEG and EMG signals as inputs to CNN models, there are two commonly used methods for representing the signals as images: calculating a time–frequency domain representation of the signal to generate spectrogram images (Zhai et al., 2017; Wang et al., 2018; Xia et al., 2018; Chaudhary et al., 2019; Côté-Allard et al., 2019; Dai et al., 2019; Duan et al., 2019; Tayeb et al., 2019) or organizing the processed signals in the time domain to create signal images (Atzori et al., 2016; Schirrmeister et al., 2017; Ameri et al., 2018; Ding et al., 2018; Zia ur Rehman et al., 2018; Amin et al., 2019; Côté-Allard et al., 2019; Li et al., 2019; Tayeb et al., 2019; Zhang et al., 2019; Zhao et al., 2019; Chen et al., 2020; Tang et al., 2020; Fang et al., 2021). Note that the term image here refers merely to a CNN input and does not require the use of an image in the colloquial sense (such as a picture). For example, signal images are just the time series data reshaped into a proper CNN input (discussed further in section 2.2.2) and the convolution is actually being done on the time series signal data directly. Both methods show prominent use with EEG and EMG-based models, with neither method demonstrating an obvious supremacy when it comes to model performance. Also, since EEG and EMG have never been used simultaneously as inputs to a CNN model to classify task weight, it is unclear which image type will allow for the best fusion of EEG and EMG within the classifier. Since both image types use a different domain representation, there is a chance they may target different responses of the signal, offering different information to the CNN classifier. Spectrogram images (time–frequency domain) may trend toward representing the oscillatory behavior of the signals, while the signal images (time domain representation) may trend toward representing time-varying behavior, such as changes in amplitude. However, this is not a given, as the CNN model is free to extract information it deems relevant from the inputs, and it remains to be seen which input method will provide the best performance when classifying task weight. For these reasons, both image types (spectrogram images and signal images) will be evaluated to determine which is the most suitable method to use for EEG–EMG fusion.

To increase the number of images to use for classifier training, during image generation the signals were windowed using a 250 ms window with 50% overlap. This windowing was used for both image types, with both a spectrogram and signal image being generated for each window. A window length of 250 ms was chosen, since studies have shown that 300 ms is the maximum amount of delay a system can experience before the user becomes unable to control the device (Tang et al., 2014). Even though this study was performed offline, limiting the window length to a time that fits within the real-time delay target allows for a more realistic evaluation of the EEG–EMG-fusion-based CNN models as a potential method of control for assistive and rehabilitation robots.

#### 2.2.1. Spectrogram Images

To generate the spectrogram images, a Short-Time Fourier Transform (STFT) was calculated for each window of the EEG and EMG signals, providing a time–frequency domain representation of the signals. The time and frequency resolution of the STFT was chosen so the resulting images would be of a suitable size for use as an input to a CNN model: large enough to have an appropriate time/frequency resolution, but not so large as to require an infeasible amount of memory and computational power. Using trial and error, a spectrogram image size of 68 × 32, for each signal channel, was chosen. For the time resolution, the STFT was calculated using a Hann window with a length of 56 samples and 75% overlap, which resulted in an image width of 68 pixels (for the 4,000 Hz sampling rate of the measurement system). The frequency resolution of the STFT was chosen so that an image height of 32 pixels would be obtained for the frequency range of interest for both EEG (0.5–40 Hz) and EMG (20–500 Hz). Due to the differences in bandwidth, this meant that EEG and EMG had different STFT frequency resolutions, but their image height was kept the same to simplify their combination into a single image during fusion. The STFT was calculated across the entire frequency range of 0–4,000 Hz using an FFT size of 3,200 for EEG and 256 for EMG. Then, the images were cropped to only include the portions of the image within the respective bandwidth of each signal type. This resulted in five spectrogram images (3 EEG channels and 2 EMG channels) of size 68 × 32 for each time window.

Following image generation, the pixel values of the spectrogram images were normalized to be between 0 and 1. Due to the highly variable nature of EEG and EMG signals between subjects, and the different scale in frequency magnitudes for EEG and EMG obtained from the STFT, the images were normalized for each subject and each signal type. After all spectrogram images were calculated for one subject, the max/min frequency magnitude value for EEG and the max/min frequency magnitude value for EMG were recorded and used to normalize all spectrogram images of that respective signal type for that subject. This ensured that both EEG and EMG spectrograms were given equal proportion within the image, regardless of the differences in signal amplitude present when recording both bioelectrical signals. This also ensured that differences observed in subject recordings did not cause certain images in the dataset to be improperly scaled based on an outlier subject.

**Figure 2:**
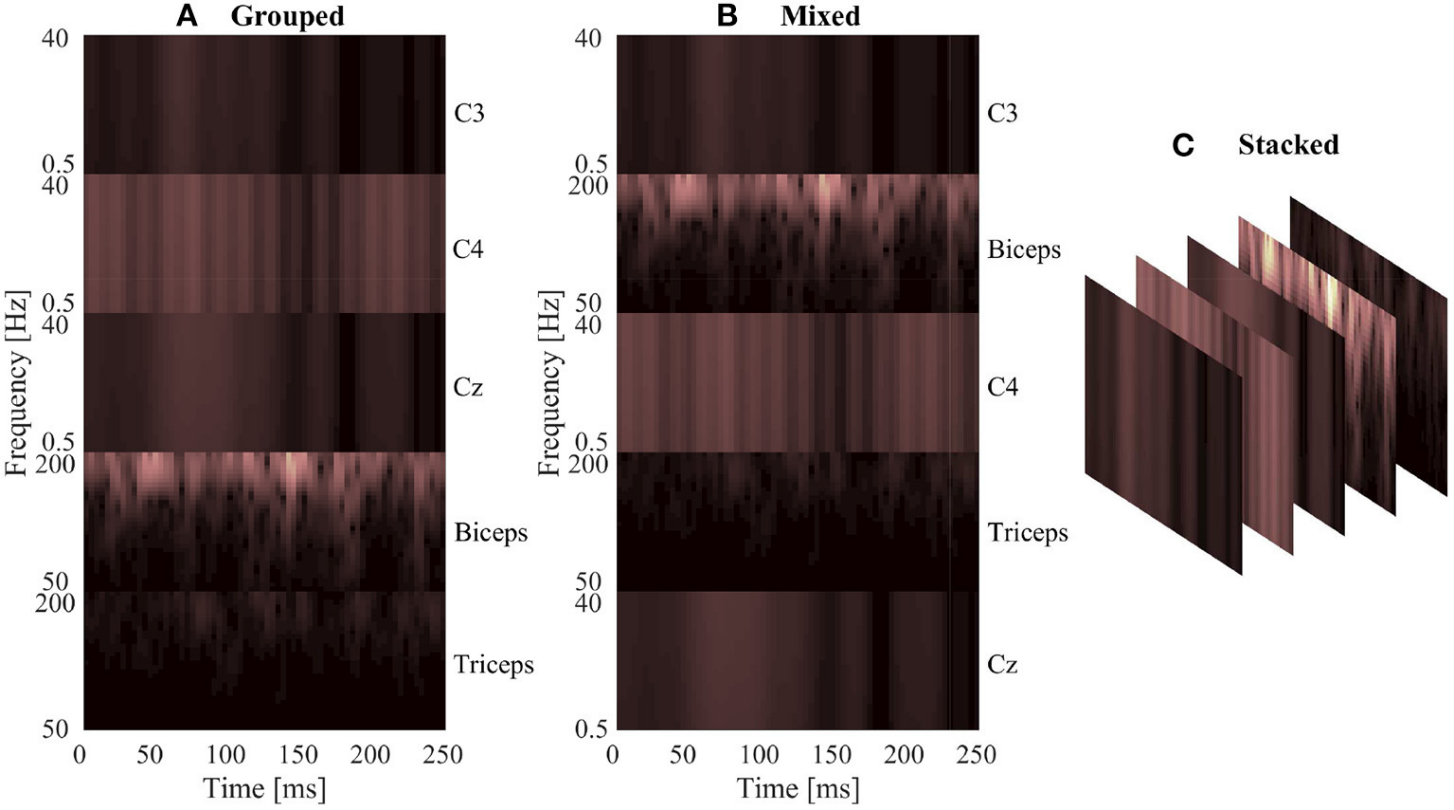
A sample normalized spectrogram image to demonstrate the three EEG–EMG fusion methods used, where **(A,B)** show single-channel spectrograms and **(C)** visualizes a multi-channel spectrogram. **(A)** Shows the grouped method, where signal channels of the same type are grouped together within the image. **(B)** Shows the mixed method, where EEG and EMG channels are alternated to mix signal types. **(C)** Provides a visualization of the stacked method, where a multi-channel spectrogram is generated by combining the different EEG/EMG spectrograms in depth-wise manner.

Once the spectrogram images had been normalized, they were combined to facilitate the fusion of EEG and EMG at the input level. Multiple methods of combining the EEG and EMG spectrogram images were performed, to investigate which method of fusing the EEG and EMG spectrogram images would provide the best model performance. In the first method of fusion (referred to here as the grouped method), the EEG and EMG spectrograms were grouped by signal type and stacked vertically to create a single 68 × 160 image comprised of the five spectrograms. The three EEG spectrograms were placed at the top of the image (in the order of C3, C4, and Cz from top to bottom) and the two EMG spectrograms were placed on the bottom of the image (in the order of biceps, then triceps from top to bottom). This fusion method grouped spectrograms of the same signal type together within the image, causing the convolution of the image to initially happen within the same signal type and only fusing the signals initially along the single border between EEG and EMG. An example of this method can be seen in Figure 2A. The second fusion method (referred to here as the mixed method) stacked images vertically once again, but this time EEG and EMG spectrograms were alternated to provide a better mix between signal types. The order from top to bottom went C3, biceps, C4, and triceps, Cz. This method of fusion provides more areas within the image were EEG and EMG will be convolved together during the initial CNN layer, since there are more borders between the EEG and EMG portions of the image. An example of this method can be seen in Figure 2B. The final fusion method (referred to here as the stacked method) stacked the images depth-wise to create a multi-channel image, similar to how a color picture will have three values per pixel location to represent levels of red, green, and blue. In this case, every pixel location contained 5 values (one for each EEG and EMG spectrogram) to result in an image with a shape of 68 × 32 × 5. An example of this method can be seen in Figure 2C. To provide a baseline comparison for evaluating the fusion methods, spectrograms containing only EEG and only EMG signal information were also generated to see if fusion can outperform using one signal alone. Two spectrogram types were generated for both EEG and EMG: vertically stacked spectrograms (68 × 96 for EEG and 68 × 64 for EMG) to provide single-channel spectrograms to compare to the grouped/mixed methods, and depth-wise stacked spectrograms (68 × 32 × 3 for EEG and 68 × 32 × 2 for EMG) to provide multi-channel spectrograms to compare to the stacked method.

#### 2.2.2. Signal Images

Conversely, generating the signal images only required the time series signals to be organized into an array to form the image, since the convolution is being performed on the time series data directly. After filtering, the five signal channels from each window were stacked vertically to create an image where the width was the number of time samples in that window, and the height was the number of signal channels. This resulted in a 1,000 × 5 image for each window, in which the pixels values of the image were the signal amplitude at that time point (in mV). The width of 1,000 resulted from the 250 ms window length with the 4,000 Hz sample rate used by the measurement system.

**Figure 3:**
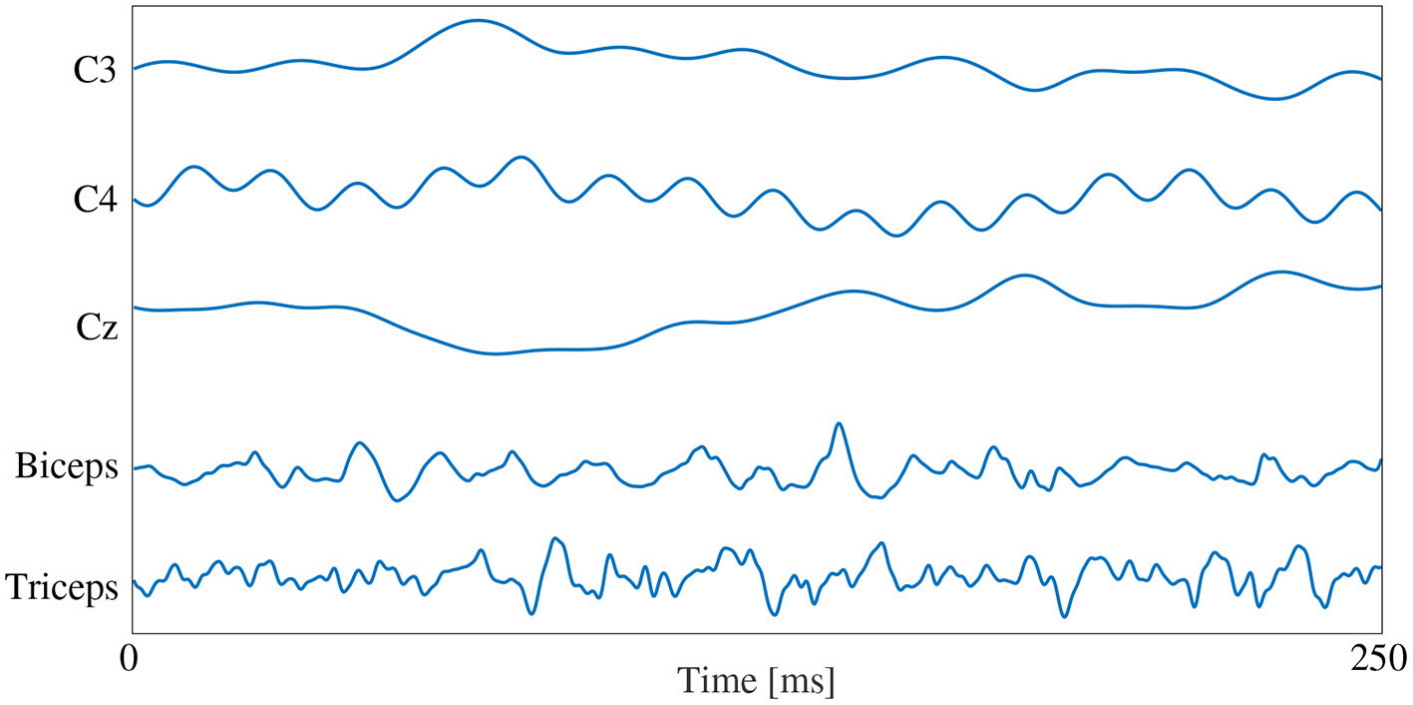
A graphical representation of a sample normalized signal image. The image height contains 5 rows, one for each signal channel, and the image width is dictated by the number of samples in each 250 ms window (1,000 samples at the 4,000 Hz sampling rate).

The signal images were normalized using the same method as the spectrogram images, by subject and by signal type. The max/min amplitude value of EEG and EMG for each subject was recorded and used to scale all signal values between 0 and 1. To account for magnitude differences between the two signal types, the EEG portion of the image was scaled using the EEG min/max and the EMG portion of the image was scaled using the EMG min/max, preventing the larger EMG values from dominating the image by diminishing the contribution of the smaller magnitude EEG signals. A graphical representation of the normalized signal image can be seen in Figure 3. Similar to the spectrogram images, signal images comprising of only EEG and only EMG were also generated to provide a comparison point for evaluating EEG–EMG fusion.

#### 2.2.3. Qualitative Image Response

**Figure 4:**
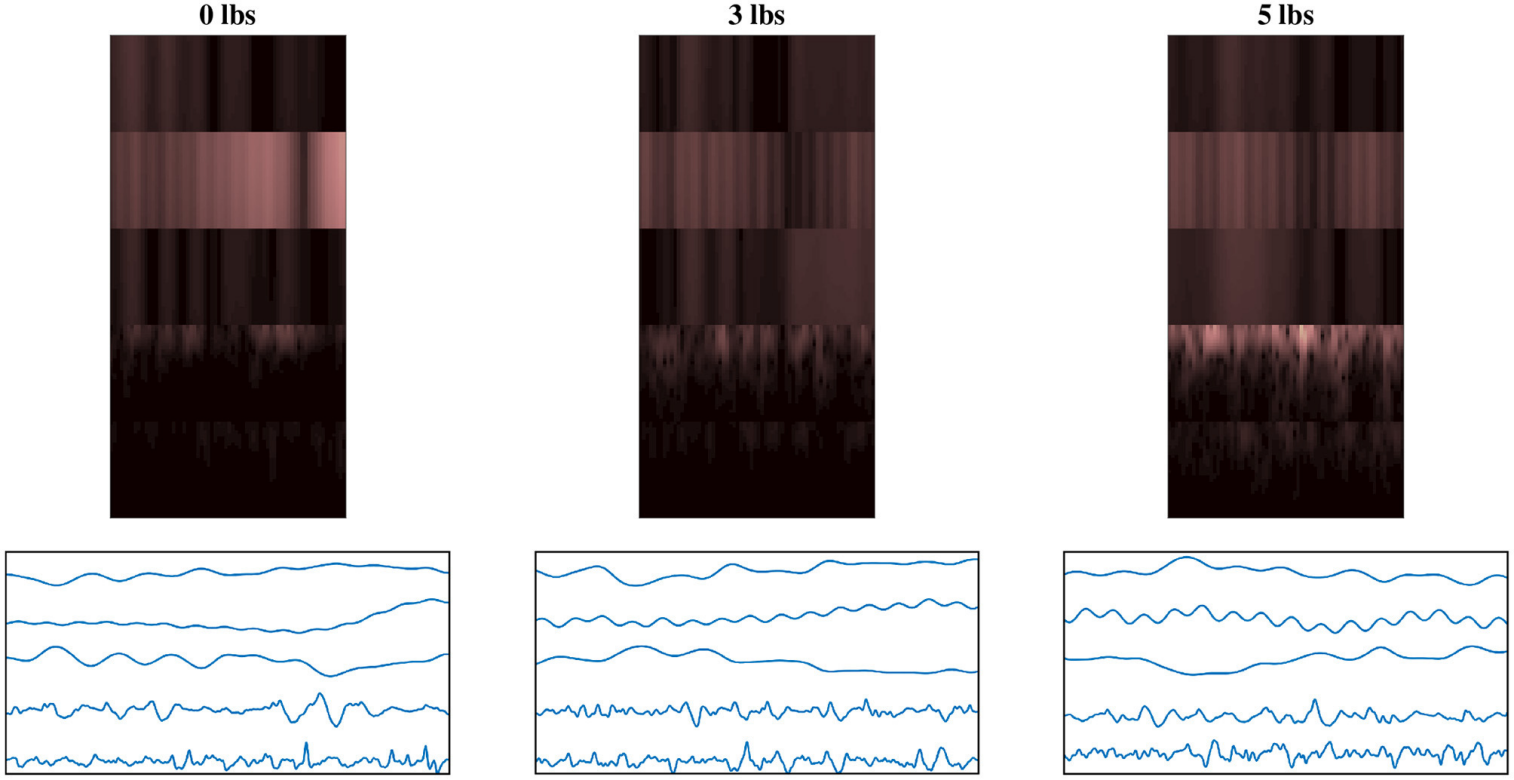
Example normalized spectrogram images and graphical representations of sample normalized signal images for each of the three weight levels, showing the qualitative variations in the images as task weight changes. During different task weights, the distribution of frequency magnitudes across time/channels is different in the spectrogram images and the shape of the time domain signal varies in the signal images. The columns each represent a different task weight level (described by the label above), with the rows being a matched spectrogram and signal image taken from the same time window. The spectrograms shown use the grouped fusion method to arrange the channels. The images shown follow the same labeling convention as the sample images shown in Figures 2, 3, excluded here to avoid clutter.

To help illustrate the response of the EEG/EMG signals during task weight changes, an example normalized spectrogram image along with a plot of the normalized signals for all three weight levels (0 lbs, 3 lbs, and 5 lbs) can be seen in Figure 4. Based on this qualitative assessment of the signal and spectrogram images, it can be seen that the images show different behavior in both the time domain and the time–frequency domain, depending on task weight. The distribution of frequency magnitudes across time/channels is different in the spectrogram images and the shape of the time domain signal varies in the signal images. This provides a qualitative demonstration that there are changing patterns within the images for different task weights, which may be able to be detected by the CNN models and used to train a classification model.

### 2.3. CNN Model Training

Once the dataset of images was developed, the CNN models based on fused EEG–EMG inputs were trained to classify task weight. Model training was done using TensorFlow 2.3.0 with Keras 2.4.3 (Chollet, 2015) in Python 3.8. The models trained were subject specific, meaning that each subject had a model trained using only their data. To accomplish this, each subject's data were split into three parts: training, validation, and testing. The first two repetitions of each speed–weight combination were dedicated as training data, while images generated from the third repetition were separated into two equally sized groups: validation and testing data. To ensure that no bias was induced by the split, the order of the windows within the third motion repetition was randomized and a stratified split was used to ensure a 50/50 division, while keeping the number of observations of each class balanced within the validation and testing set. The validation dataset was used during model optimization while the testing set was kept separate until the final model evaluation, in order to reduce potential for model bias and overfitting.

Model training had two stages. First, the base configuration of the model was determined (via trial and error) to determine design factors such as number of layers, batch size, and optimizer choice, among others. The base configuration used for each model type was the same for all subjects and is discussed further in sections 2.3.1 and 2.3.2. Once a base configuration for the model had been determined, the second stage of training was to tune the model further using hyperparameter optimization. This tuning focused on finding optimal parameter values for the setting of the layers within the set base model design. The structure of the model (e.g., number of layers, types of layers used, etc.) was not changed during this optimization, only select hyperparameter values were updated. Using Keras-Tuner 1.0.1 (O'Malley et al., 2019), the values of select hyperparameters were tuned using the Random Search optimization method to find the set that resulted in the best validation performance. The search space checked 50 random combinations of hyperparameters, and trained each combination twice to account for variance in model training. Using the validation dataset, the hyperparameters were evaluated and the set that resulted in the lowest validation loss was selected as the final hyperparameters to use for model training. Bayesian optimization was also tested as a potential method for hyperparameter tuning, but it was found to result in a slight reduction in performance compared to the Random Search method, so it was not used during training of the final models. Early Stopping (using a patience value of five and an epoch limit of 50) was also implemented into model training, using Keras, to stop classifier training once improvements were no longer seen in the validation loss of the model. This was done to prevent overfitting and to speed up training time. All models were optimized and trained using batch size of 32, which was found using trial and error. Categorical Cross-Entropy was used as the loss function with Adaptive Moment Estimation (ADAM) being used as the optimizer for all model types. A Stochastic Gradient Decent (SGD) optimizer was also tested, but it resulted in a reduction in accuracy and longer training times, so ADAM was chosen instead. The hyperparameters being tuned, and their range of possible values, were the same for all subjects; however, each subject had their own hyperparameter optimization performed to adjust the models better to the behavior seen in their specific EEG and EMG signals. The hyperparameters that were tuned for each model type can be seen in Table 2 and are discussed further in sections 2.3.1 and 2.3.2.

**Table 2:** The hyperparameters tuned during optimization, with the range of possible values used by the Random Search algorithm.

**Hyperparameter**	**Parameter values**
Kernel size (Spectrogram)	3×3, 5×5, (third layer only) 7×7
Kernel width (Signal)	3–55 (step size of 2)
Filters	8, 16, 32, 64, 128, 256, 512, 1,024
Dropout %	0.0–0.5 (step size of 0.05)
Units (FC Layers)	20–500 (step size of 20)
ADAM learning rate	10^−5^–10^−2^ (logarithmic sampling)

*Unless specified (in brackets next to the hyperparameter name), all hyperparamters and value ranges shown were used for all model types. Two exceptions to this are the kernel size for the stacked models, which were limited to 3 × 3 to account for the smaller image size, and the split convolution filter, which did not include the 1,024 filter setting to prevent an out of memory error while training*.

#### 2.3.1. Spectrogram CNN Models

**Figure 5:**
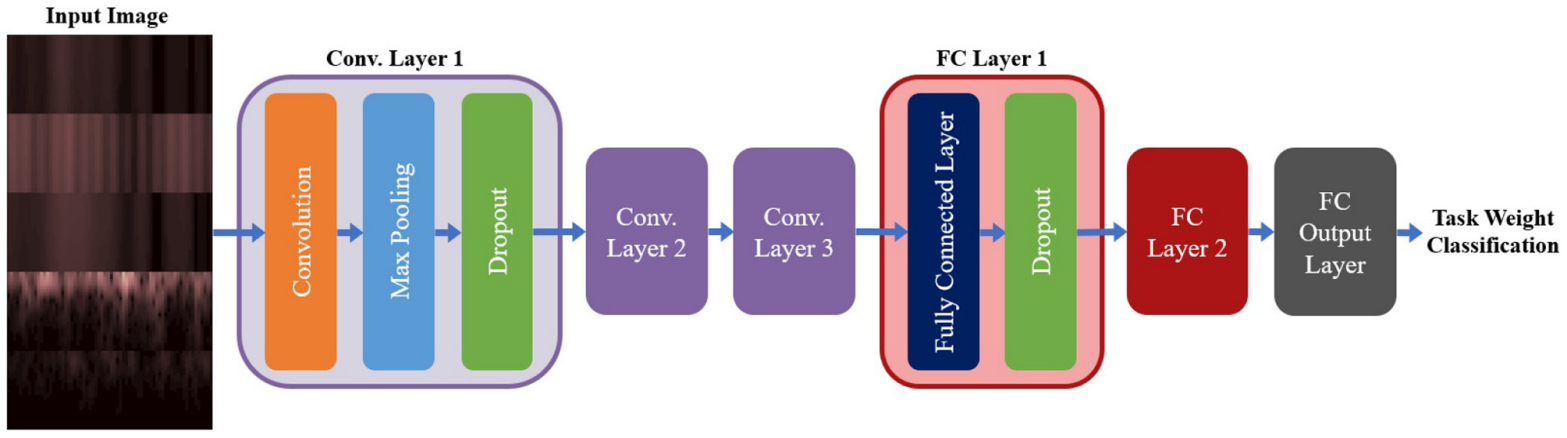
The base model configuration used for all three spectrogram CNN model types. All spectrogram model types used three convolution layers, followed by two FC layers and an output FC layer to perform the final classification. Each convolution layer had three sub-layer steps (convolution, max pooling, and dropout) and each FC layer had two sub-layer steps (the FC step followed by dropout). Note, that repeated layers only show the sub-layers for the first layer, to reduce redundancy and condense the diagram.

A summary of the base model configuration for the spectrogram models can be seen in Figure 5. The base configuration for the spectrogram CNN models consisted of three convolution layers followed by two Fully Connected (FC) layers, with a third FC layer used to output the class probabilities. All convolution was done used valid padding, a stride of 1 × 1 and the Rectified Linear Unit (ReLu) for the activation function. Each convolution layer included three sub-layer steps: convolution, followed by a max pooling layer (with a size and stride of 2 × 2), and then a dropout layer to reduce overfitting. Both FC layers contained two sub-layers: the FC step, followed by a dropout layer. Batch Normalization was tested as an alternative to using dropout for the convolution layers, but it led to a reduction in accuracy so it was not used. The output FC layer used a softmax activation function to perform classification. This configuration was used for both the single-channel and multi-channel models (as well as their EEG and EMG only equivalents); the only difference between model types being the size of the inputted image. The hyperparameters chosen for tuning, and the range of values included in the search space, are shown in Table 2. Note that these are the same for both model types except for one deviation: the kernel size. For the multi-channel models, the kernel size was fixed at 3 × 3. This was to account for the smaller image size being fed into the model; with certain combinations of larger kernels, the tensor that was passed between convolution layers could be reduced below the minimum allowable size, causing an error in model training.

#### 2.3.2. Signal CNN Models

**Figure 6:**
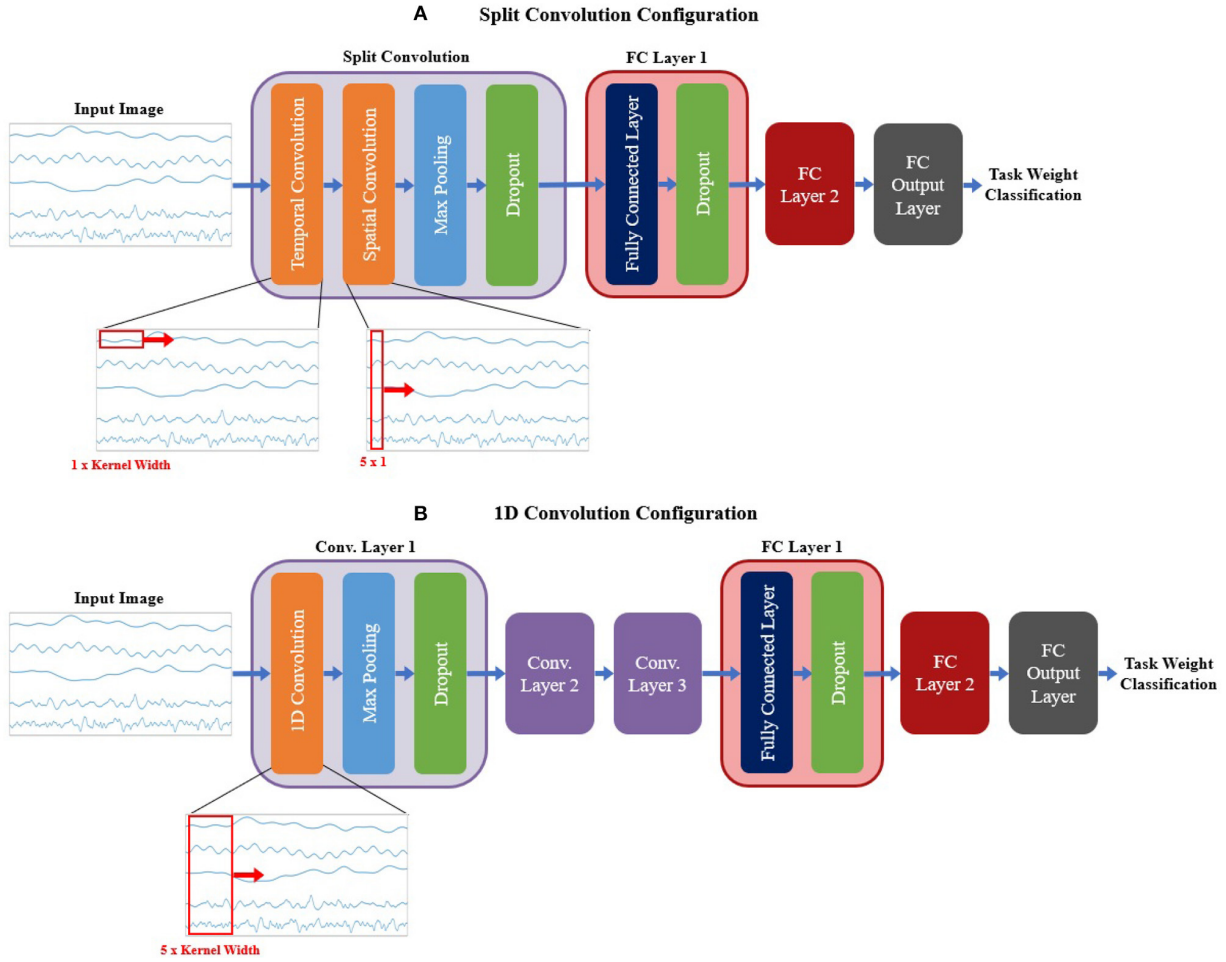
The base model configurations used for the **(A)** split convolution and **(B)** 1D convolution models. Visual representations of the differences between both convolution types are shown in the expanded view below each diagram, detailing the changes in kernel size used to facilitate both types of convolution. Split convolution used one split convolution layer comprised of temporal and spatial convolution sub-layers, followed by a max pooling and dropout sub-layer. 1D convolution used three convolution layers, each with three sub-layer steps (convolution, max pooling, and dropout). All signal model types followed convolution with two FC layers (containing two sub-layer steps: the FC step followed by dropout) and an output FC layer to perform the final classification. Note, that repeated layers only show the sub-layers for the first layer, to reduce redundancy and condense the diagram.

For the signal CNN models, two base configurations were tested, shown in Figure 6. The first configuration employed a method commonly used when developing CNN models based on time domain signal inputs for EEG (Schirrmeister et al., 2017; Amin et al., 2019; Li et al., 2019; Zhao et al., 2019), referred to here as split convolution. The name arises from that fact that it takes the first convolution layer and splits it into two back-to-back convolution steps. This method sets the kernel size of the first two convolution layers such that convolution is only happening across one axis of the image at time, with Layer 1 having a kernel size of 1 × kernel width (to only convolve temporally across the time axis of the image) and Layer 2 having a kernel size of image height × 1 (to only convolve spatially across signal channels). The output of the temporal convolution layer is fed directly into the spatial convolution layer, with both layers using valid padding, stride of 1 × 1, and ReLu for the activation function. The output of the temporal convolution layer is fed into a max pooling layer (with a size and stride of 1 × 2), followed by a dropout layer. This is followed up by two FC layers (both using ReLu as the activation function and a dropout sub-layer), then a third output FC layer using a softmax activation function to perform the final classification. A summary of the base model configuration for the split convolution signal model can be seen in Figure 6A.

The second base configuration tested for the signal-image-based CNNs used regular one dimensional (1D) convolution layers to train the models. Unlike the split convolution, this layer type convolves across both the time and signal channel axis simultaneously as it moves across the time axis of the image (for this reason only a kernel width is specified, since all signal channels are always included in the convolution). This is a common method of using CNNs for time series signals, so it is useful to see how it compares to the split convolution method commonly seen in the EEG literature. This configuration was similar in makeup to the spectrogram base configuration (except using 1D convolution instead of 2D convolution), comprising of three convolution layers followed by two FC layers and a third FC layer for classification. All convolution layers used valid padding, a stride of 1 and ReLu for the activation function. Each convolution layer followed up the convolution step with a max pooling layer (with a size and stride of 2) then a dropout layer to reduce overfitting. Both FC layers used a dropout layer after the FC step. The output FC layer used a softmax activation function to perform the final classification. A summary of the base model configuration for the 1D convolution signal model can be seen in Figure 6B.

Both signal image model types used similar hyperparameter tuning settings; however, there were slight variations between them to account for the differences in the configurations. Due to an out-of-memory error while training, the split convolution models could not use a filter setting of 1,024 and was limited to 512 as the maximum number of filters for any one convolution layer. For both model types, the hyperparameters chosen for tuning, and the range of values included in the search space, are shown in Table 2.

### 2.4. Model Evaluation

Once the optimized models for each subject were trained, they were evaluated to assess the performance of CNN-based EEG–EMG fusion. To achieve this, the withheld test data for each subject were inputted to their final models to obtain predictions about what task weight was being held during each test image. Since three task weights were used during data collection (0 lbs, 3 lbs, and 5 lbs), each classifier was trained to output a three-class prediction, where each output label corresponded to one of three task weights. This output was compared with the actual class label to obtain an accuracy score for each model. This accuracy was then averaged across all subjects to obtain an overall accuracy score for each fusion method, which was then used to compare performance via statistical analysis (performed using IBM SPSS 27). First, the merits of each fusion method were evaluated by comparing EEG–EMG fusion to using EEG and EMG alone. The accuracy scores for each fusion method were compared to the accuracy scores of the EEG/EMG only models of the same model type to see if the increase in accuracy obtained via EEG–EMG fusion was statistically significant. A one-way Within-Subjects Analysis of Variance (ANOVA), followed by pairwise comparisons with the Bonferroni *post-hoc* test, was performed on the accuracy scores for the models of each type (four one-way ANOVAs in total). Separate ANOVAs were used for each model type to account for the different number of models present, depending on the type (the single-channel spectrogram model type contained 4 models, because of the use of both the grouped and mixed fusion methods, while the other model types only contained three models each). This prevents model type from being a factor for a two-way ANOVA, so separate one-way ANOVAs were used instead. Following this, the methods of EEG–EMG fusion were compared to each other using a one-way Within-Subjects ANOVA, (using the Bonferroni *post-hoc* test for pairwise comparisons) to determine if statistically significant differences exist between the accuracy obtained from each fusion method. The purpose of this was to see if any particular EEG–EMG fusion method provided a clear advantage in regard to classification accuracy.

To evaluate the robustness of each model further, the effect of movement speed on accuracy was also evaluated. The classifier output predictions were separated depending on the speed at which the movement was being performed, and accuracy was calculated separately for the fast and slow movement speed groups. Since changes in movement speed during dynamic motion can greatly affect bioelectrical signals, it is important to know how the CNN EEG–EMG fusion models will perform in the presence of such variability. To be useful in the control of robotic devices, the models need to be able to operate adequately during the different speeds required to perform various rehabilitation and assistance tasks. To see if the effect of speed was statistically significant, a two-way Within-Subjects ANOVA was performed on the speed-separated accuracies for each model type. Similar to the model accuracy one-way ANOVA, the two-way ANOVA was performed between models of the same type, resulting in four two-way ANOVAs in total. Note, for all statistical tests performed (on both the overall model accuracy and the speed specific accuracy), a significance threshold of *p* < 0.05 was used.

As a final analysis of model performance, the class predictions from every subject were combined and used to plot a confusion matrix for each CNN model. This was done to observe how the models performed for each task weight and to further verify that the classifiers were adequately trained. To evaluate the model fitting of each classifier further, the confusion matrices were used to calculate the class-wise precision (the likelihood that a class prediction is correct) and recall (the likelihood that all observations of a specific class are correctly classified) scores, to check the balance between both metrics.

## 3. Results

### 3.1. Model Accuracy

**Figure 7:**
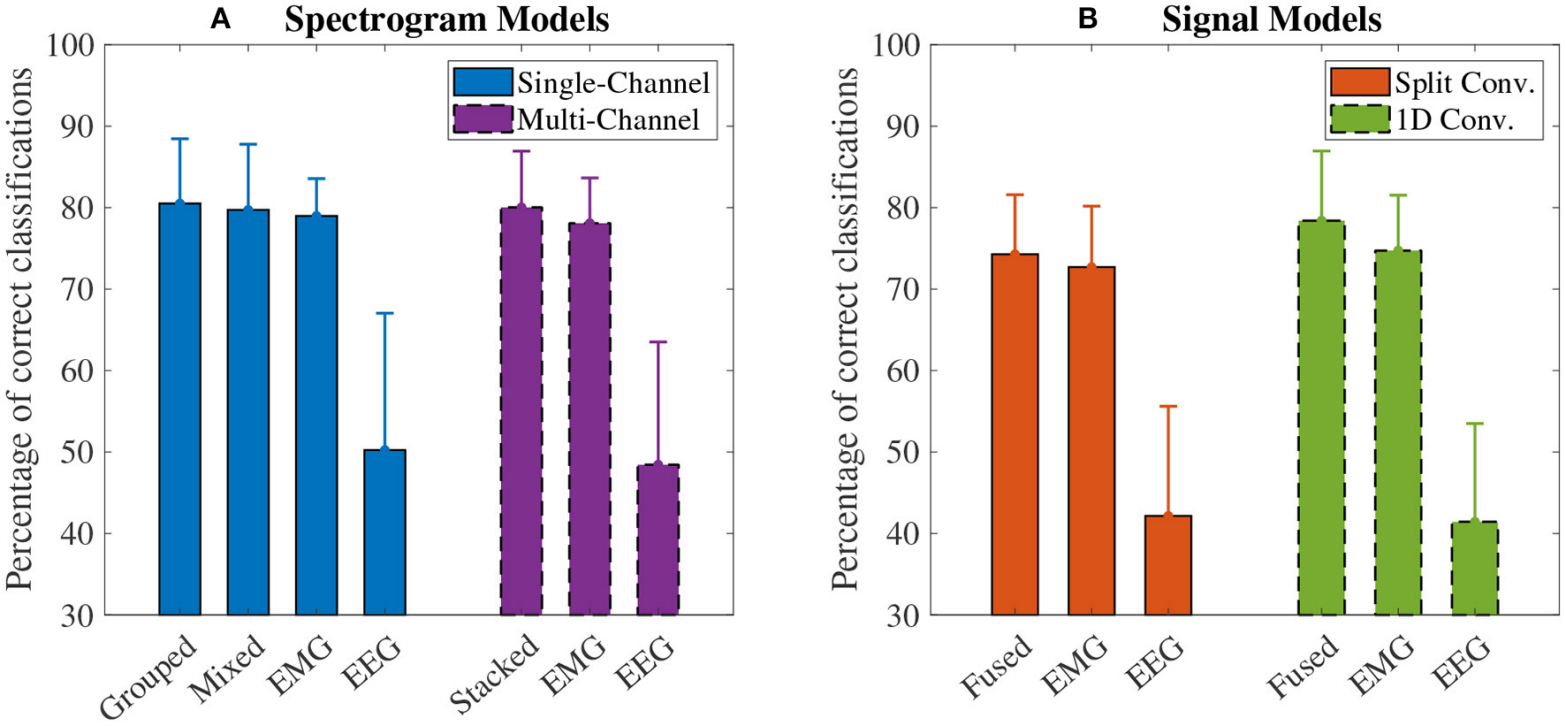
The mean accuracy of all **(A)** spectrogram and **(B)** signal based CNN models, calculated across both speeds and all task weights. Error bars represent one standard deviation. Note that the *y* axis begins at 30% (chance level for these models is 33.33%).

The accuracy results for the spectrogram-based CNN models are summarized in Figure 7A. For all models, the mean accuracy was above chance level (33.33%). The highest accuracy was obtained by the grouped fusion method (80.51 ± 8.07%). This was higher than the other single-channel models, beating the EEG (50.24 ± 17.06%, *p* < 0.001) and mixed fusion method (79.72 ± 8.19%, *p* = 0.025) models, and trending toward a higher mean accuracy than EMG (78.98 ± 4.66%, *p* = 1.000), but the difference between these two was not statistically significant. The next highest performing spectrogram model was the stacked fusion method (80.03 ± 7.02%), which outperformed the multi-channel EEG model (48.44 ± 15.32%, *p* < 0.001), and trended toward a higher accuracy than the multi-channel EMG model (78.09 ± 5.65%, *p* = 0.382), but again this increase in accuracy was not statistically significant. The stacked fusion method also showed a higher mean accuracy than all other single-channel models (except for the grouped fusion method). When comparing the spectrogram fusion methods to their equivalent EEG/EMG model types, the increase in accuracy for all fusion models was statistically significant for EEG, but not EMG; however, a clear trend did emerge, where mean accuracy increased when using EEG–EMG fusion.

The accuracy results for the signal-based CNN models are summarized in Figure 7B. Again, all models showed a mean accuracy higher than chance level. The highest accuracy was observed from the 1D convolution EEG–EMG fusion model (78.40 ± 8.70%), which showed a statistically significant increase in accuracy over using EEG alone (41.44 ± 12.25%, *p* < 0.001), but not EMG alone (74.73 ± 6.90%, *p* = 0.054), even though the trend is toward an increase in accuracy. The split convolution EEG–EMG fusion model (74.28 ± 7.42%), while lower than 1D convolution fusion, also showed a statistically significant improvement over using only EEG (42.16 ± 13.67%, *p* < 0.001), but not EMG (72.70 ± 7.60%, *p* = 0.401); however, as with 1D convolution, the mean accuracy tends to increase between the split convolution fusion and EMG only models. When comparing the signal fusion methods to their equivalent EEG/EMG model types, the increase in accuracy for both fusion models was statistically significant for EEG, but not EMG; however, once again a trend did emerge where mean accuracy increased when using EEG–EMG fusion.

For comparing the EEG–EMG fusion methods of all model types together, the results of the pairwise comparisons can be seen in Table 3. The mean accuracy for split convolution was found to be statistically significantly lower than all other fusion methods, indicating that it is the worst performing method of fusion. The difference in accuracy between grouped and mixed fusion was also found to be statistically significant, meaning that grouped fusion performed better than mixed within this sample group. Stacked, grouped, and 1D convolution fusion showed no statistical significance in their accuracy differences, meaning that these methods demonstrate similar performance within this sample group. In general, there was a trend of spectrogram-based methods having a higher mean accuracy than signal-based methods (which held true for both EEG–EMG fusion, as well as EEG and EMG alone).

**Table 3:** The *p* values obtained from the pairwise comparisons in the one-way ANOVA comparing the accuracy of the different CNN based EEG–EMG fusion methods.

**Fusion method**	**Grouped**	**Mixed**	**Stacked**	**Split Conv**.	**1D Conv**.
Grouped	-	**0.041**	1.000	**<0.001**	0.431
Mixed	**0.041**	-	1.000	**0.003**	1.000
Stacked	1.000	1.000	-	**<0.001**	1.000
Split Conv.	**<0.001**	**0.003**	**<0.001**	-	**0.018**
1D Conv.	0.431	1.000	1.000	**0.018**	-

*Statistically significant values (p < 0.05) are shown in bold*.

### 3.2. Speed Specific Accuracy

**Figure 8:**
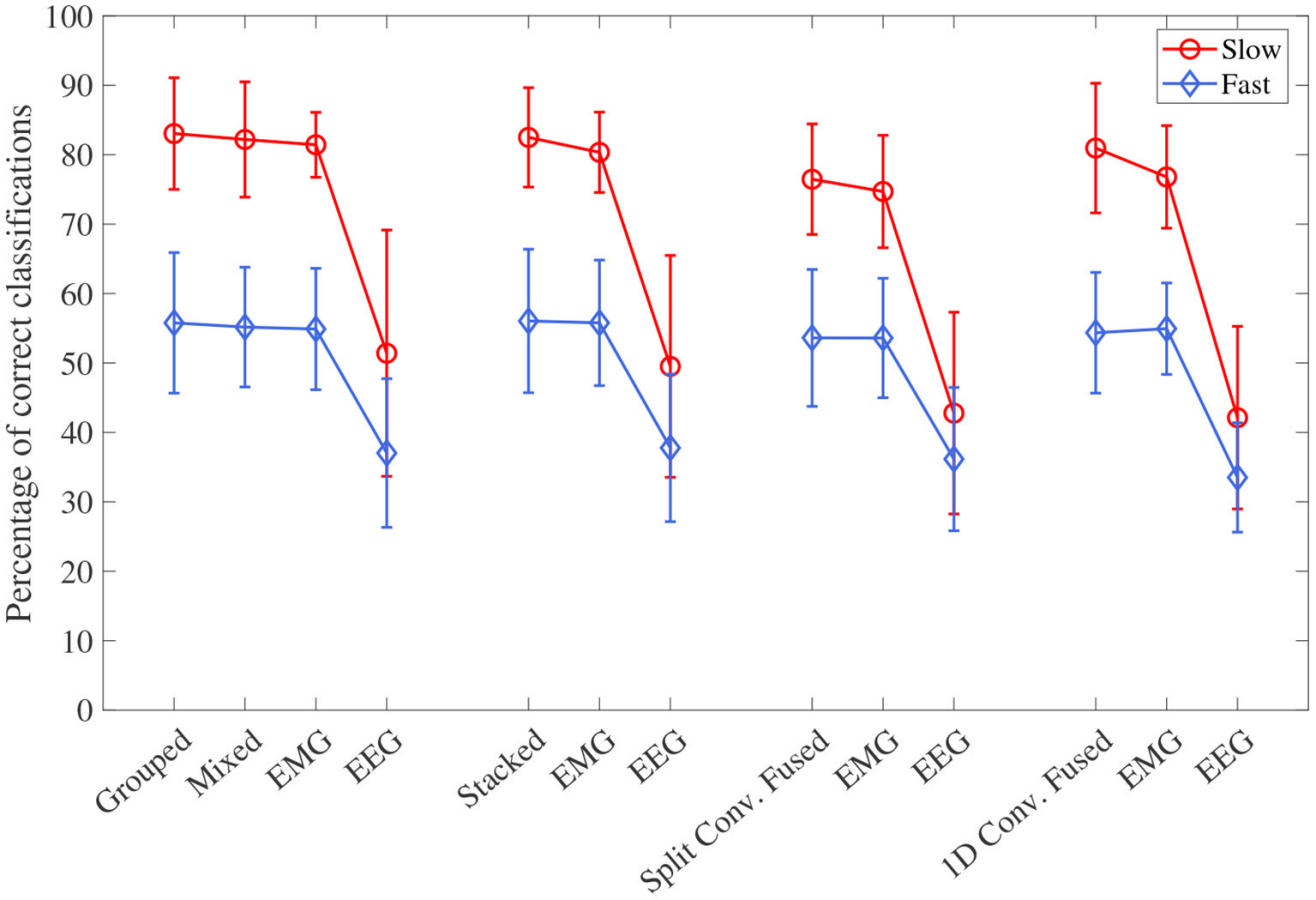
The mean accuracy for all CNN models, separated by the two speed levels (fast and slow). Models of the same type are grouped together, with the order of the groups from left to right as follows: single-channel spectrogram models, multi-channel spectrogram models, split convolution signal models, and 1D convolution signal models. Error bars represent ± one standard deviation.

The accuracy results, separated into the fast and slow speed groups, can be seen in Figure 8. For all four model types, the effect of speed was statistically significant (*p* < 0.001 for all). Looking at the plot, it can be seen that performance was significantly worse during the fast speed for all models. All models still remained above the chance level during the fast motion speed; however, EEG accuracy decreased almost to this point (with 1D convolution in particular being essentially at the chance level). It can also be seen that, even when accounting for speed, the trend of EEG–EMG fusion outperforming EEG and slightly increasing accuracy over EMG still remained; however, the increase was much less during fast motion (and in the case of 1D convolution, EMG alone was slightly higher than fusion during the fast speed).

### 3.3. Classifier Performance

**Figure 9:**
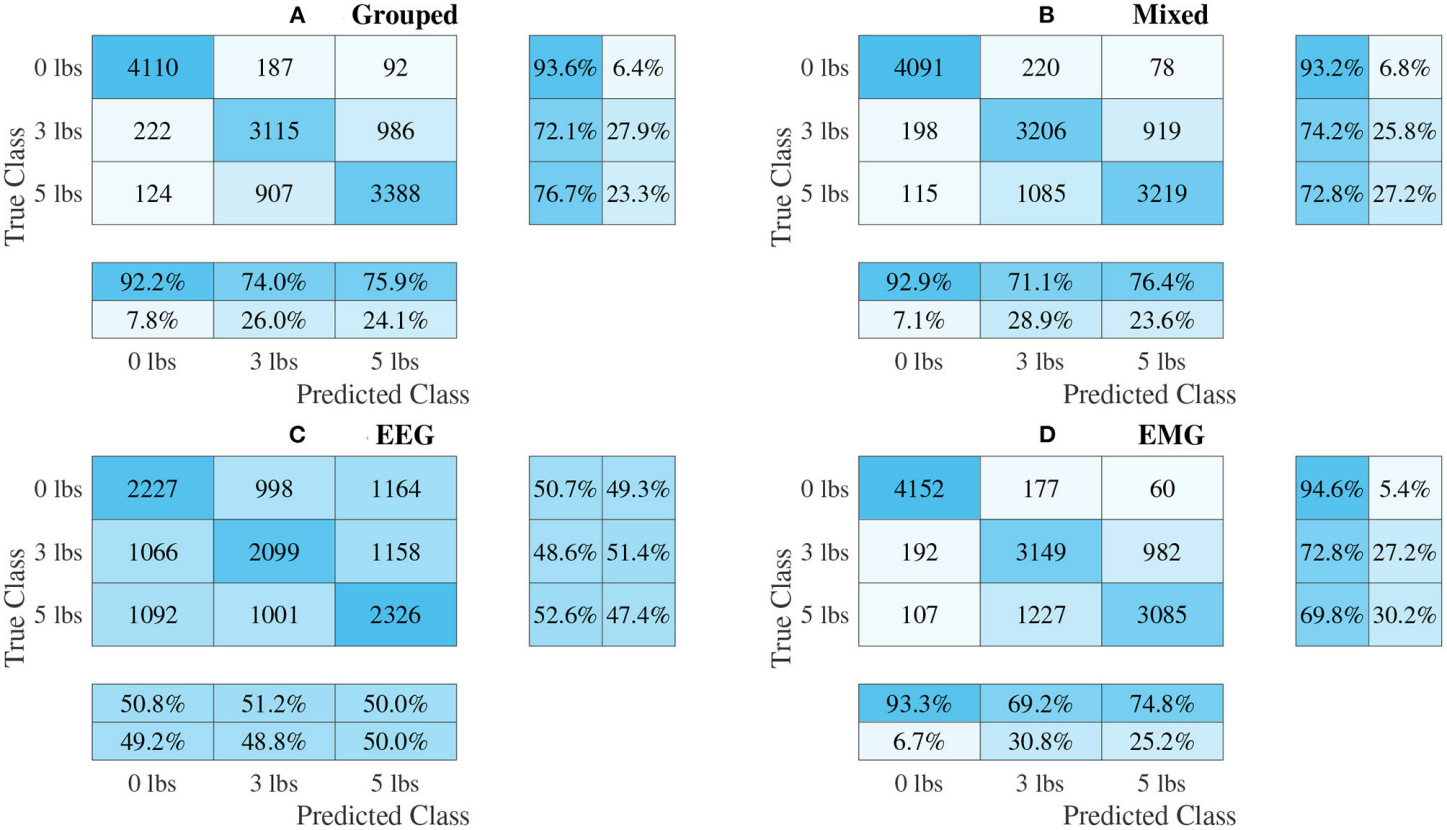
Confusion matrices, using the combined classification results for all subjects, for the single-channel spectrogram-based CNN models. **(A)** Shows the matrix for the grouped fusion method while **(B)** shows the matrix for the mixed fusion method. **(C,D)** Show the matrices for the EEG and EMG only models, respectively. Each matrix contains a positive/negative precision score summary in the final two rows, and a positive/negative recall score summary in the final two columns.

**Figure 10:**
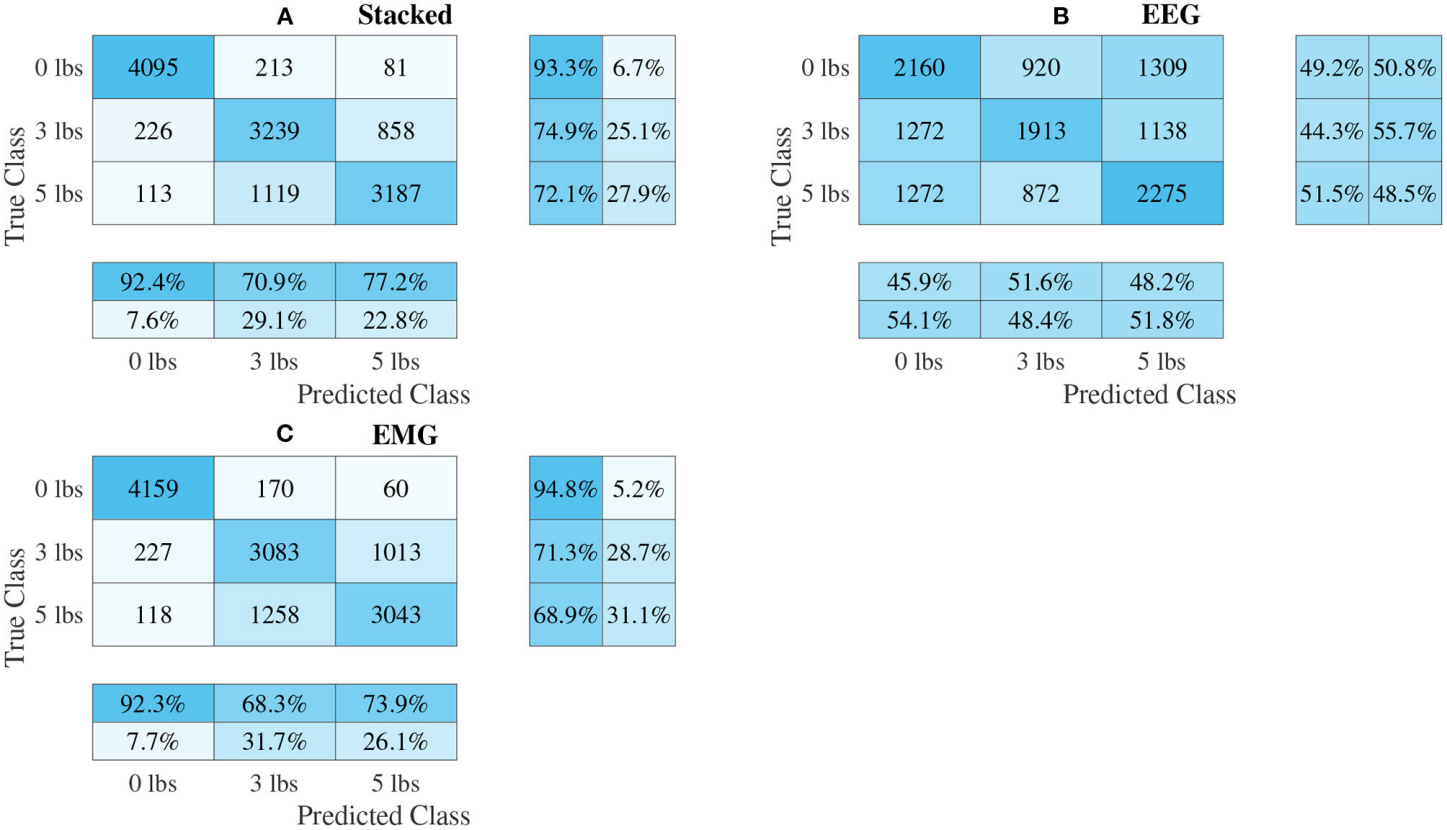
Confusion matrices, using the combined classification results for all subjects, for the multi-channel spectrogram-based CNN models. **(A)** Shows the matrix for the stacked fusion method, while **(B,C)** how the matrices for the EEG and EMG only models, respectively. Each matrix contains a positive/negative precision score summary in the final two rows, and a positive/negative recall score summary in the final two columns.

**Figure 11:**
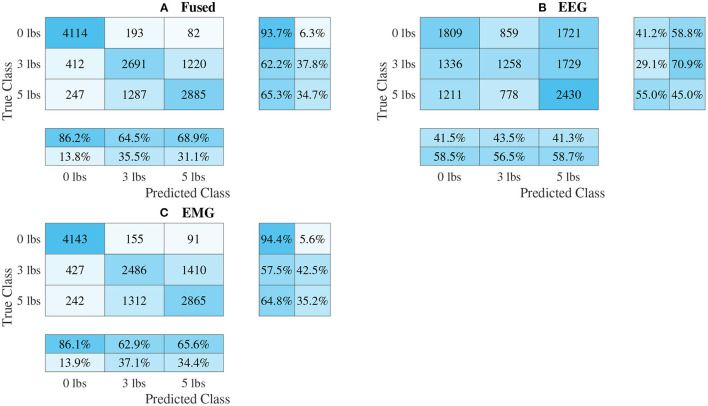
Confusion matrices, using the combined classification results for all subjects, for the split convolution signal-image-based CNN models. **(A)** Shows the matrix for the EEG–EMG fusion model, while **(B,C)** how the matrices for the EEG and EMG only models, respectively. Each matrix contains a positive/negative precision score summary in the final two rows, and a positive/negative recall score summary in the final two columns.

**Figure 12:**
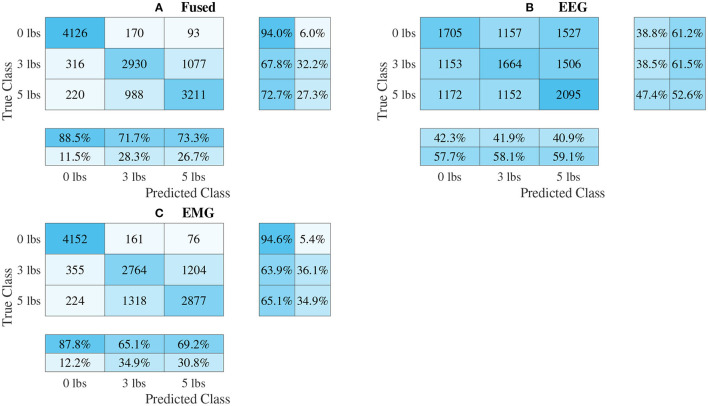
Confusion matrices, using the combined classification results for all subjects, for the 1D convolution signal-image-based CNN models. **(A)** Shows the matrix for the EEG–EMG fusion model, while **(B,C)** how the matrices for the EEG and EMG only models, respectively. Each matrix contains a positive/negative precision score summary in the final two rows, and a positive/negative recall score summary in the final two columns.

The confusion matrices for all four model types can be seen in Figures 9–12, with each figure corresponding to one type of model. For each model type, a confusion matrix is presented for every model (fusion, EEG, and EMG), shown as sub-figures. Looking at the class outputs, it can be seen that all models successfully classified 0 lbs at a much higher rater rate when compared to 3 and 5 lbs (which were similar to each other in performance). An exception to this trend is the two signal-based EEG models (shown in Figures 11B, 12B for split and 1D convolution, respectively), which had generally poor performance for all weight classes. The precision and recall scores for the spectrogram-based models are relatively similar between the two metrics, demonstrating that on average the fit of the models was balanced in its performance. The signal-based models show less balance between the two metrics comparatively, although not to a large degree.

## 4. Discussion

The goal of this study was to evaluate if CNNs could be used as a new method of input level EEG–EMG fusion to classify task weight during dynamic elbow flexion–extension motion. The hope was that the CNN's ability to automatically learn relevant information from an inputted image may capture aspects of the EEG–EMG relationship not yet found when using manual feature extraction techniques. To this end, this study investigated several methods of representing the EEG–EMG signals as images (to convert the bioelectrical signals into a form suitable for input into a CNN), as well as ways to fuse EEG/EMG during convolution while in image form. This was done to act as a preliminary analysis of these methods, to see which CNN-based EEG–EMG fusion techniques show the most promise to justify their further development. This will ultimately benefit the field of rehabilitation and assistive robotics by providing a new method of EEG–EMG fusion that can be used by the control system of such devices to detect user tasks to adapt accordingly, resulting in devices that are safer and more comfortable to control.

Looking at the model accuracy for each method, it can be seen that all models performed above the chance level (33.33%), and that the precision/recall scores were relatively balanced between the two metrics (albeit less so for the signal-based models than the spectrogram models). This shows that the CNN classifiers were successfully able to decode task weight information from the EEG/EMG signals, indicating that this classification method is feasible for this task. When comparing EEG–EMG fusion to using EEG or EMG alone, a clear trend is seen where using EEG–EMG fusion improves the performance of the models. For all model types, EEG–EMG fusion resulted in some level of accuracy improvement, as well generally higher precision and recall scores (and for the classes where the precision/recall scores were not higher, they were almost the same). Even though no statistically significant difference was found between EEG–EMG fusion and using EMG alone, this does not completely invalidate the use of this new method. Despite the current iteration of these models showing that the improvements gained from using EEG–EMG fusion compared EMG are small, the fact that improvements are consistently observed when using fusion demonstrates that the method shows potential as a tool to improve task weight classification and should be investigated further. By focusing future work on developing improvements to model performance, the accuracy gains of using EEG–EMG fusion may be increased, providing a stronger justification for its use over EMG alone. Based on the trend, it is highly likely that increasing study power through the recruitment of more subjects may result in the difference in accuracy becoming statistically significant. Also, improving the quality of the EEG signals may improve the EEG–EMG fusion models further. Looking at the EEG models, a clear drop in accuracy and classifier performance can be seen when compared to EMG and EEG–EMG fusion, which is likely due to the noisy nature of EEG signals. Due to their significantly smaller signal amplitude, EEG is more prone to signal contamination from motion artifacts and magnetic interference when compared to EMG, which can make it harder to use for classification. The use of more advanced noise rejection techniques and better measurement hardware may improve EEG task weight classification, which should in turn improve the EEG–EMG fusion models. Increasing the amount of EEG channels being used may also help improve the EEG models, as well as EEG–EMG fusion, since it will allow the classifier to draw from more sources from different areas in the brain. However, this trade-off needs to be balanced when using this application for wearable robotics, as these devices are very limited in the hardware resources available. Even though EEG showed worse performance compared to EMG, it was still clearly able to be of some benefit to the EEG–EMG fusion models, since their mean accuracy always tended to be higher than the models based on EMG alone. As a preliminary analysis of EEG–EMG fusion, this work was able to demonstrate that there is a clear benefit to using CNN-based EEG–EMG fusion over just using EEG or EMG alone. It showed a trend of CNN-based EEG–EMG fusion resulting in an increase in mean accuracy, demonstrating the feasibility of these methods and providing a justification for their continued development. Future work should focus on improving these models further to increase the improvements that these techniques provide.

Another objective of this work was to see which methods of combining EEG/EMG would result in the best performance when using CNN models. Looking at the accuracy results of each fusion method, it is clear to see that the CNNs models did perform differently depending on the method used. Of all the fusion methods, split convolution using signal images as inputs performed the worst (and this difference was found to be statistically significant when compared to all other model types). Even though other studies have used this method successfully for classification when only using EEG signals (Schirrmeister et al., 2017; Amin et al., 2019; Li et al., 2019; Zhao et al., 2019), it is clear from this work that it is not suitable when used with EEG/EMG together for task weight classification. For signal-image-based models, using a traditional 1D convolution to perform CNN-based EEG–EMG fusion results in better performance. For the spectrogram-image-based models, it was less obvious which fusion type is superior. The grouped method had the highest mean accuracy, and the increase over the mixed method was statistically significant, which implies that of the two ways to mix EEG/EMG spectrograms, using the grouped method is better. Between grouped and stacked methods though, the difference in accuracy was not statistically significant, so it is less clear which method is best. It should be noted that the stacked spectrogram method is much more computationally efficient than the grouped method (CNNs can perform convolution on a smaller image with multiple channels faster than a larger image with only one channel), which may be a reason to use the stacked method. Since both methods have similar accuracy, the faster method is more ideal, as the end goal of these models is to be used in real time in wearable robotic exoskeletons. Regardless, both methods should be developed further in future work to investigate which method is ultimately superior. Comparing between spectrogram-image-based models and signal-image-based models, it can be seen that, in general, the mean accuracy of spectrogram models was higher. This is also confirmed when looking at the confusion matrices, as the precision and recall scores are not as balanced for the signal models. This even held true for the EEG and EMG only models, in particular EEG, which showed a significant drop in accuracy (as well as precision and recall) for the signal models. This makes sense, since it is well known that much of the relevant information related to motor tasks is encoded in the frequency of the EEG signals (Vaid et al., 2015). It is likely that the time-domain-based representation of the signal images was not able to capture this information as well as the time–frequency-based representation used in the spectrogram images could. This, in turn, would also affect the EEG–EMG fusion methods, which are drawing information EEG, as well as EMG. Despite the lower mean accuracy, no statistically significant difference was found between the 1D convolution, grouped, and stacked methods. This means that even though the trend would make it seem like the 1D convolution method is worse, it should still be considered for future development. One potential benefit of the 1D convolution method is that it requires fewer processing steps to generate the images. Performing a calculation like a STFT can be comparatively time consuming, and computationally expensive, so the use of signal-image-based models may be justified when used in a real-time context for a wearable robotic system. The slight decline in model performance may be outweighed by the efficiency provided by the simpler method; however, further testing and development is needed to confirm this. Since the purpose of this experiment was to investigate the initial feasibility of the different CNN-based EEG–EMG fusion methods, an extensive evaluation of the computational complexity of each algorithm was not performed. The discussion here is based merely on qualitative observations; however, next steps should focus on additional quantitative evaluations of model complexity, which will become essential for moving the models toward a real-time application when integrating them into a wearable device. Ultimately, all three fusion methods (grouped, stacked, and 1D convolution) should continue to be improved and investigated, since there was not one method shown to definitely have better performance and all three methods have clear benefits.

The models can be evaluated further by looking at the speed separated results, as well as the confusion matrices, to examine how robust the classifiers are to changes in task weight and motion speed. Looking at the confusion matrices in Figures 9–12, it can be seen that task weight affected classification accuracy. All models were able to recognize the 0 lbs class at a much higher rate than the 3 lbs and 5 lbs classes. While both of these classes still had relatively good precision and recall scores, 3 lbs and 5 lbs were often misclassified as each other, but not 0 lbs, which implies that the models had a harder time distinguishing the smaller difference in weight. This still does present some level of benefit to a wearable robotic exoskeleton, since even knowing that the user is holding something or not, could be useful for allowing the control system to adapt; however, future work should look at improving the model results further to make them more consistent across different task weights. It is clear from Figure 8 that speed also has a great effect on performance for all models, with the fast speed having a significantly lower accuracy than the slow speed. The EEG–EMG fusion models were still above chance level when moving at the fast speed, which means that they are still able to recognize relevant patterns in the EEG/EMG signals, just not as effectively. It also should be noted that the trend of EEG–EMG fusion having higher accuracy than using EEG or EMG alone continued, even when separated by speed; however, the increase was very small during fast speed (and the 1D convolution model was actually slightly less accurate than EMG during fast motion). There are multiple things that may be causing this phenomenon. First, faster movements are more likely to cause the EEG and EMG signals to be corrupted by motion artifacts. The more aggressive movements performed by the subject during the fast motion speed may be causing more motion artifacts, which in turn makes the signals harder to use for classification. To alleviate this, more advanced filtering techniques should be used during signal processing to remove this noise. The second reason why the fast motion may be harder to classify is due the nature of task weight classification itself. Despite being related to muscle force (a heavy weight needs more muscle force to move), the task weight itself is not actually a direct measurement of muscle force. The muscle force required to perform an elbow flexion–extension repetition will be a combination of the speed at which the subject was moving and the weight they are holding. It is possible that this is causing smaller weights, moving at a faster speed, to have the appearance of a larger weights at a slower speed, causing the misclassification. EMG in particular may be prone to showing this pattern, since EMG is a measurement of muscle activation. This theory is supported by the authors' previous work, which classified task weight using EEG–EMG-fusion based on traditional machine learning techniques that rely on manual feature extraction. In this study, it was found that all EEG–EMG fusion models showed a statistically significant improvement in accuracy when adding a feature for speed information (in this case a categorical label for fast and slow), seeing improvements of 1.2% for the best performing fusion method (Tryon and Trejos, 2021). Basic knowledge about the speed of the motion given to the classifier was enough to help improve accuracy, so it stands to reason this could be possible for the CNN models as well. Future work should investigate ways to include speed information into the input of the CNN, and evaluate the effect that this has on classifier performance. Finally, the reduction in accuracy seen during the fast motion trials could be due to the way the CNN models fit to the data. The nature of how the EEG/EMG signals were windowed mean that there are more observations of movement during the slow speed than the fast speed (since for slow motion it took longer to complete an elbow flexion–extension repetition, and there were the same number of repetitions for both speeds). It is possible that the models became fitted more heavily toward the slow speed data points, causing poorer performance for the fast speed. To account for this, future work should look at collecting more repetitions for the fast motion speed to balance out the classifier training.

Based on the results of this work, CNN-based EEG–EMG fusion has shown to be a feasible method for classification of task weight, and warrants further development to increase the improvements provided by this technique. One potential area for improvement is in the dataset used to train the models. As previously discussed, increasing the number of subjects may improve study power and allow for more statistically significant results; however, this can also allow for the development of generalized models that do not need to be subject specific. Ideally, to allow for ease of use, a wearable robotic exoskeleton should be able to function for any user with minimal training/calibration required. With a large enough sample of the population, general classification models can be pre-trained so that new users can skip the time consuming step of classifier training. An improved dataset can also benefit subject specific models by collecting more elbow flexion–extension repetitions, as well as more combinations of speed and weight. One aspect of CNN models is that their performance can be reduced for smaller training datasets (Luo et al., 2018), so collecting more data per subject should improve performance. More speed/weight combinations will help to provide a more in-depth analysis of the robustness of the classifiers, and will improve their functionality. Since this was the first analysis of CNN-based EEG–EMG fusion, only a small range of weights (0lbs to 5 lbs) and two speeds (approximately 10°/s and 150°/s) were evaluated. It is possible that the inclusion of more task weights, and a larger range of allowable dynamic motion speeds, will affect the classifier performance further, so this effect should be investigated in future work. The current task weight resolution of the classifiers (three weight levels) may limit their use for assistance with daily-living tasks, where the user is unpredictability lifting many objects of varied weights; however, this resolution could still be relevant for more controlled tasks, such as rehabilitation. During rehabilitation exercises, the movement patterns and weight changes performed by the user will be more predictable than activities of daily living, making the use of these classifiers more feasible. The models developed for this work could be used to help the control system of a wearable robotic rehabilitation device automatically adapt changing weights as the user performs different exercises, and will not require the user/therapist to enter the weight change manually, via some external input method, which may feel cumbersome for the user (for example a smartphone app). The ultimate goal, however, is to keep improving the CNN-based EEG–EMG fusion models to increase their resolution, making them a viable tool for use in many different applications, such as assistance with daily tasks.

One method that may improve CNN-based EEG–EMG fusion is to increase the complexity of the models via the inclusion of other deep learning architectures into the model configurations. One popular example of this is the development of models that combine CNNs with Long Short-Term Memory (LSTM) classifiers. LSTM models are beneficial for the classification of information that changes over time, by retaining a memory of inputs (Greff et al., 2017). Since the behavior of EEG and EMG signals will change depending on what stage of elbow flexion–extension motion is currently being evaluated (for example the biceps muscle should be more dominant during flexion), LSTMs may benefit the model by being able to incorporate this information better than using only a CNN. Other studies have shown that CNNs, combined with LSTMs, can be used for EEG (Ditthapron et al., 2019; Zhang et al., 2019; Wilaiprasitporn et al., 2020) and EMG (Xia et al., 2018) classification, and LSTMs alone have been used during decision-level EEG–EMG fusion (Tortora et al., 2020), so there is evidence to suggest that this can be a beneficial technique for improving EEG/EMG models. Future work should evaluate the use of combined CNN–LSTM models for input-level EEG–EMG fusion. Another potential way of improving CNN-based EEG–EMG fusion is to explore other methods of calculating time–frequency signal images. While the STFT is a popular time–frequency representation method, it is far from the only one. Other studies have shown that Wavelet-Transform-based images can also work for EEG (Chaudhary et al., 2019; Xu et al., 2019) and EMG (Côté-Allard et al., 2019) CNN models, so future work should investigate these methods as an alternative to using STFT spectrograms for CNN-based EEG–EMG fusion. Improving these models will move them closer to being practically implemented within a wearable robotic exoskeleton, where they can improve the usability of these devices during rehabilitation and assistive tasks.

## 5. Conclusion

This work demonstrated the feasibility of using CNNs as a method of input level EEG–EMG fusion for task weight classification during dynamic elbow flexion–extension. It presents a new EEG–EMG fusion method that can be used to improve the performance of bioelectrical signal controlled robotic devices for assistance and rehabilitation. During the experiment performed, it was shown that a trend exists where EEG–EMG fusion resulted in a higher mean accuracy compared to using EEG and EMG alone. Different methods of representing the EEG/EMG signals for use in the CNNs were also evaluated, and it was found that time–frequency-image-based models (spectrograms) tended to outperform time domain (signal) models; however, signal models using 1D convolution may still have the potential to match spectrogram model performance. Future work should expand upon the results shown here, and focus on improving performance by increasing model complexity through the inclusion of other deep learning architectures (such as Long Short-Term Memory networks), as well as, investigating other time–frequency image representation methods (such as Wavelet Transforms). It should also focus on improving the training dataset used by collecting EEG/EMG signals during more speed/weight combinations, collecting more motion repetitions from each subject, and collecting data from a larger population of subjects, to allow for a more in-depth analysis of model robustness, as well as better trained models. Using CNNs to facilitate EEG–EMG fusion presents a new way to utilize these bioelectrical signals for the control of wearable robotic devices, and implementing EEG–EMG fusion for task weight classification will allow such devices to adapt to changes in system dynamics so that they can perform assistive and rehabilitation tasks in a more stable and robust way. This will ultimately improve the user experience, leading to safer devices that can be more widely adopted as a new treatment and assistance solution for musculoskeletal disorders.

## Data Availability Statement

The datasets presented in this article are not readily available because the Human Research Ethics Board at Western University has not given approval to share the data collected for this study. Requests to access the datasets should be directed to atrejos@uwo.ca.

## Ethics Statement

The studies involving human participants were reviewed and approved by the Human Research Ethics Board at Western University (Project ID: 112023). The patients/participants provided their written informed consent to participate in this study.

## Author Contributions

JT and ALT contributed to conception and design of the study. JT performed the data collection, model development, results analysis, and wrote the first draft of the manuscript. All authors contributed to manuscript revision, read, and approved the submitted version.

## Funding

This research was funded by the Natural Sciences and Engineering Research Council (NSERC) of Canada under grant RGPIN-2020-05648, by the Canadian Foundation for Innovation (CFI) and the Ontario Research Fund (ORF) under grant 35152, and by the Ontario Ministry of Economic Development, Trade and Employment, and the Ontario Ministry of Research and Innovation through the Early Researcher Award (ER14-10-159).

## Conflict of Interest

The authors declare that the research was conducted in the absence of any commercial or financial relationships that could be construed as a potential conflict of interest.

## Publisher's Note

All claims expressed in this article are solely those of the authors and do not necessarily represent those of their affiliated organizations, or those of the publisher, the editors and the reviewers. Any product that may be evaluated in this article, or claim that may be made by its manufacturer, is not guaranteed or endorsed by the publisher.
